# A mouse model reproducing the pathophysiology of neonatal group B streptococcal infection

**DOI:** 10.1038/s41467-018-05492-y

**Published:** 2018-08-07

**Authors:** Elva Bonifácio Andrade, Ana Magalhães, Ana Puga, Madalena Costa, Joana Bravo, Camila Cabral Portugal, Adília Ribeiro, Margarida Correia-Neves, Augusto Faustino, Arnaud Firon, Patrick Trieu-Cuot, Teresa Summavielle, Paula Ferreira

**Affiliations:** 1 ICBAS—Instituto de Ciências Biomédicas de Abel Salazar, Universidade do Porto, 4150-313 Porto, Portugal; 2 i3S—Instituto de Investigação e Inovação em Saúde, Universidade do Porto, 4200-135 Porto, Portugal; 3 IBMC—Instituto de Biologia Molecular e Celular, Universidade do Porto, 4200-135 Porto, Portugal; 4 ESS—Escola Superior de Saúde, Instituto Politécnico do Porto, 4200-072 Porto, Portugal; 5 UMIB—Unit for Multidisciplinary Investigation in Biomedicine (Endocrine, Cardiovascular & Metabolic Research), University of Porto, 4150-313 Porto, Portugal; 6 Life and Health Sciences Research Institute (ICVS), School of Medicine, University of Minho, 4710-057 Braga, Portugal; 7 ICVS/3B’s, PT Government Associate Laboratory, 4710-057 Braga/4805-017, Guimarães, Portugal; 8 Division of Infectious Diseases, Department of Medicine Solna, Karolinska Institutet, 171 76, Stockholm, Sweden; 9 Institut Pasteur, Unité de Biologie des Bactéries Pathogènes à Gram-positif, Centre National de la Recherche Scientifique (CNRS ERL 6002), Paris, 75015 France

## Abstract

Group B streptococcal (GBS) meningitis remains a devastating disease. The absence of an animal model reproducing the natural infectious process has limited our understanding of the disease and, consequently, delayed the development of effective treatments. We describe here a mouse model in which bacteria are transmitted to the offspring from vaginally colonised pregnant females, the natural route of infection. We show that GBS strain BM110, belonging to the CC17 clonal complex, is more virulent in this vertical transmission model than the isogenic mutant BM110∆cylE, which is deprived of hemolysin/cytolysin. Pups exposed to the more virulent strain exhibit higher mortality rates and lung inflammation than those exposed to the attenuated strain. Moreover, pups that survive to BM110 infection present neurological developmental disability, revealed by impaired learning performance and memory in adulthood. The use of this new mouse model, that reproduces key steps of GBS infection in newborns, will promote a better understanding of the physiopathology of GBS-induced meningitis.

## Abstract (web summary)

Animal models of group-B streptococcal infections are needed to develop effective therapies. Here, Andrade et al. present a mouse model in which the bacteria are transmitted from vaginally colonised pregnant females to their offspring, causing neonatal meningitis and neurological developmental disabilities.

## Introduction

Neonatal bacterial meningitis is a severe life-threatening disease and a major cause of neurological disability worldwide. Group B *Streptococcus* (GBS) remains the leading cause of severe invasive infections among neonates and, together with *Escherichia coli*, account for at least two-thirds of all deaths from neonatal meningitis^1,2^. GBS is a commensal organism of the genitourinary and/or gastrointestinal tract of adult humans and has been isolated from vagina and/or rectum of 15–40% of pregnant women^2^. Maternal colonisation and transmission to foetus and newborns is the most common cause of neonatal GBS infections leading to pneumonia, septicaemia and meningitis^2^.

The current prevention strategy of *intrapartum* antibiotic prophylaxis (IAP), for parturient at risk of GBS transmission to newborns, has reduced the cases of pneumonia and septicaemia^2,3^. The incidence rates of GBS meningitis have remained relatively stable over the past 20 years^4,5^ but now appear to be increasing^6,7^. Furthermore, maternal colonisation is not affected by IAP treatment^3^ and the overall mortality rate for GBS neonatal infections remains at approximately 10%. As a consequence, morbidity rates associated with meningitis caused by GBS infection has not changed substantially over decades^2,8^ remaining unacceptably high. In addition, up to 50% of surviving infants experience neurodevelopmental impairment (NDI)^2,9–11^, highlighting the inefficacy of current treatment and the urgent need for new preventive and/or therapeutic approaches. The mechanisms that lead to the devastating outcome of GBS-induced meningitis are not elucidated. Clinical data concerning neonatal meningitis are limited as this disease is difficult to diagnose due to subtle and nonspecific symptoms^8,12^, and it is estimated that more than 30% of the cases are asymptomatic^13,14^. Moreover, protocols in which only neonates with confirmed bacteraemia are evaluated for meningitis result in missed diagnoses, as blood cultures may be negative in 15–38% of cases^12^. Thus, a better understanding of the pathogenesis and pathophysiology of GBS meningitis must be gained. To this end, mice and rat models of GBS disease have been developed, but they often target a particular step of the pathophysiological process (organ colonisation, septicaemia, meningitis) and although they have generated important knowledge, they also have the potential of yielding misleading information. This is particularly the case when non-natural infection routes, such as intraperitoneal, subcutaneous, or intracerebral GBS inoculation, that by-pass the mother-to-newborn transmission and the normal bacteraemia-meningitis sequence in the neonate, are used^15–19^. Moreover, the use of irrelevant animal models in research results in a lower likelihood of translation to the clinic^20,21^. Thus, an animal model in which the disease induced closely resembles GBS natural infection in humans is still missing.

Here, we present a mouse model that recapitulates the GBS newborn infection pathogenesis by enabling vertical transmission of the pathogen from vaginally colonised pregnant females to their progeny. We validate this model by using the hypervirulent GBS strain BM110, a serotype III strain belonging to the hypervirulent clonal complex 17 (CC17)^22–24^. In addition, the attenuated isogenic mutant BM110∆cylE that does not express the pore-forming toxin β-hemolysin/cytolysin (β-h/c) was used. This toxin is an important virulent factor^25–27^ whose overexpression leads to preterm labour in pregnant nonhuman primates^28^.

Our data show that neonatal mice born from mothers colonised with the hyper-virulent GBS strain exhibit enhanced mortality and lung pathology, which correlates with higher bacterial load. Importantly, newborns that survive to BM110 infection experience permanent NDI, as observed in humans. Thus, this mouse model, which mimics the human pathophysiology of GBS diseases, should allow a better understanding of the pathophysiology of GBS meningitis and open new avenues towards the identification of new therapeutic and neuroprotective strategies.

## Results

### β-h/c expression favours colonisation of mouse vagina

**Fig. 1:**
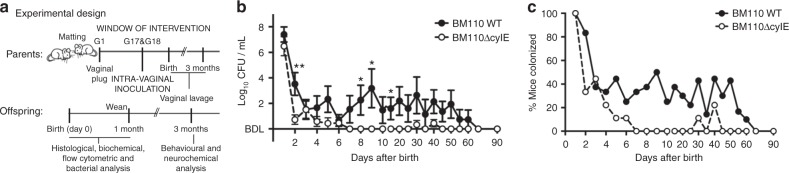
Vaginal GBS colonisation of female mice. **a** A schematic illustration of the colonisation model is shown. Pregnant BALB/c mice were intra-vaginally inoculated with 3 × 10^4^ CFU of GBS BM110 WT or BM110ΔcylE at gestational days 17 and 18. **b** After birth, GBS vaginal colonisation levels were determined at the indicated time-points in vaginal lavage by enumerating onto selective media GBS colony-forming units (CFU) [mean ± SEM, *n* = 10 (BM110 WT) and *n* = 9 (BM110ΔcylE)]. Comparisons by two-way ANOVA, with Sidak’s multiple comparison. *P < 0.05, **P < 0.01. **c** The percentage of mice remaining colonised after birth is shown [*n* = 10 (BM110 WT) and *n* = 9 (BM110ΔcylE)]

Knowing that human neonates acquire the bacterium by vertical transmission, pregnant BALB/c mice were inoculated intra-vaginally (i.vag.) with the serotype III hypervirulent strain CC17 BM110 or the isogenic attenuated strain BM110∆cylE lacking haemolytic/cytolytic activities. After several attempts, days 17 and 18 of gestation (G17 and G18) were defined as the specific window to administer the bacteria (Fig. 1a). Vaginal colonisation was monitored upon delivery by vaginal lavage and plating of recovered bacteria (the delivery day was excluded due to excess of blood and body fluids). At day one after birth, and with both GBS strains, the vaginal mucosa of all females presented high bacterial load enabling the vertical transmission of the bacterium (Fig. 1b,c). Thereafter, the bacterial levels started to decrease, but more abruptly with BM110∆cylE mutant than with BM110 WT (Fig. 1b, c). After day 6 upon delivery, the levels of bacteria remained almost exclusively below the detection threshold for the attenuated strain, while a transient and intermittent colonisation was observed with the hyper-virulent strain. At day 60, no bacterium was detected in the vaginal mucosa of all females in either group (Fig. 1b, c). Data from individual progenitors are depicted in Supplementary Fig. 1a and b.

### β-h/c expression induces a severe pathology to neonatal mice

**Fig. 2:**
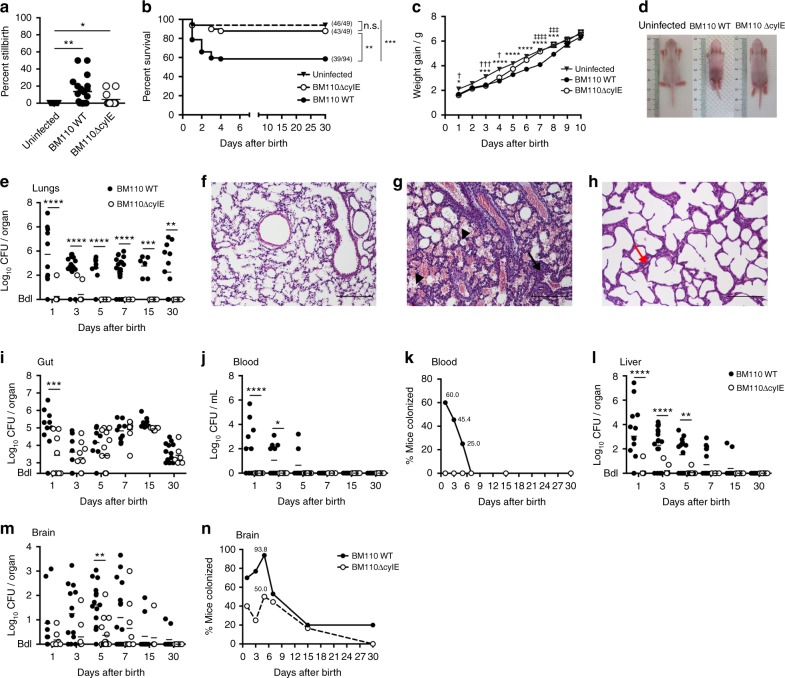
GBS vertical transmission. Pregnant BALB/c mice were intra-vaginally inoculated with 3 × 10^4^ CFU of GBS BM110 WT, BM110ΔcylE, or PBS (uninfected) at gestational days 17 and 18. **a** Percentage of term stillbirths born from indicated dams. Each symbol indicates data from a single mouse [mean, *n* = 15 (uninfected), *n* = 20 (BM110 WT) and *n* = 11 (BM110ΔcylE)]. Comparisons by one-way ANOVA, with Sidak’s multiple comparison. *P < 0.05, **P < 0.01. **b** Kaplan–Meier survival curve of neonatal mice, monitored for 30 days. Numbers in parentheses represent surviving pups versus total born [*n* = 49 (uninfected), *n* = 94 (BM110 WT) and *n* = 49 (BM110ΔcylE)]. Comparisons with log-rank (Mantel-Cox) test. **P < 0.01, ***P *<* 0.001 and n.s., not significant. **c** Body weight gain of mice following birth [mean ± SEM, *n* = 22 (uninfected), *n* = 34 (BM110 WT) and *n* = 12 (BM110ΔcylE)]. * represents the comparison between BM110 WT and uninfected pups, † BM110ΔcylE and uninfected pups, and ‡ BM110 WT and BM110ΔcylE pups. Comparisons by two-way ANOVA, with Sidak’s multiple comparison. *,^†^*P* < 0.05, ***,^†††,‡‡‡^*P* < 0.001 and ****,^‡‡‡‡^*P* < 0.0001. **d** Overall appearance of an offspring born from uninfected (left), BM110 WT- (middle), or BM110ΔcylE-colonised (right) progenitors at PND7. **e**, **i**, **j**, **l**, **m** GBS counts in lungs (**e**), gut (**i**), blood (**j**), liver (**l**), and brain (**m**) in the offspring born from BM110 WT- or BM110ΔcylE-colonised progenitors at different time points after birth. Each symbol indicates data from single pups [mean, *n* = 11 (PND1), *n* = 21 (PND2 and 7), *n* = 9 (PND3), *n* = 13 (PND15) and *n* = 13 (PND30) for BM110 WT; *n* = 8 (PND1), *n* = 9 (PND2 and 7), *n* = 15 (PND3), *n* = 6 (PND15 and 30) for BM110ΔcylE]. Comparisons by two-way ANOVA, with Sidak’s Multiple comparison. *P < 0.05, **P < 0.01, ***P < 0.001 and ****P < 0.0001. **f**–**h** H&E staining of pulmonary tissue from uninfected (**f**), BM110 WT- (**g**), or BM110ΔcylE- (**h**) colonised progenitors, at PND1. Representative micrographs are shown. Black arrow indicates interstitial inflammation and areas of atelectasis. Arrowheads indicate oedema and severe haemorrhage. Red arrow indicates mild atelectasis areas. Scale bar, 100 μm. **k**, **n** Percentage of neonatal mice whose blood (**k**) and brain (**n**) contained GBS at the indicated time points

As GBS is a major cause of stillbirth, we monitored the foetal death in pregnant mice colonised either with the hyper-virulent or the attenuated GBS strain. Stillborn accounted for 14 or 5% of all pups born from BM110 or BM110∆cylE-colonised females, respectively, while none was observed in non-colonised mothers (Fig. 2a). Following delivery, approximately 40% of the pups born from BM110-colonised females died after birth, 21% during the first 24 h of life, while 12% of pups born from BM110ΔcylE-colonised mothers died after birth, 6% in the first 24 h (Fig. 2b). In the non-colonised control group, only 6% of the pups died during the first 24 h of life (Fig. 2b). No death was recorded after postnatal day (PND) 4 in all groups of mice (Fig. 2b).

Measurement of weight gain, a sensitive marker of neonatal well-being, was recorded during the first 10 days of life. Body weight gain was reduced in pups born from GBS-colonised progenitors compared to those born from non-colonised mothers (Fig. 2c, d). This reduction was more pronounced with the pups from BM110 WT- colonised mothers (8 days) compared to pups from BM110∆cylE-colonised mothers (4 days) (Fig. 2c).

As pneumonia frequently heralds early stages of neonatal GBS disease, we investigated bacterial load and signs of lung inflammation, or loss of structure, in pups born from colonised mothers. Higher and sustained GBS bacterial loads were detected in the lungs of pups born from BM110 WT-colonised progenitors compared to those born from BM110ΔcylE-colonised mothers (Fig. 2e). These bacterial loads were correlated with the observed lung pathology (Fig. 2f–h). Indeed, neonates born from BM110 WT-colonised mothers displayed significant lung pathology with the presence of areas of atelectasis lung inflammation with moderate neutrophil infiltration in the alveoli and interstitium, narrowed airway lumen, oedema and severe haemorrhage. In contrast, pups born from BM110ΔcylE-colonised mothers showed reduced lung inflammation with mild atelectasis and mild increased thickness of alveolar wall (Fig. 2f–h). Gut GBS colonisation was also quantified and only at PND1, the pups born from BM110-colonised mothers presented higher levels of bacteria than those born from BM110ΔcylE-colonised mothers (Fig. 2i). Thereafter, no significant differences were observed between pups born from hyper-virulent or attenuated GBS-colonised females with both groups presenting high levels of GBS (Fig. 2i).

As this bacterium can disseminate into the bloodstream, GBS was also quantified in the blood of pups born from colonised mothers. GBS were present in the blood of 60% of pups born from BM110-colonised progenitors, reaching a maximum at PND1, but were no longer detected at PND7 (Fig. 2j, k). In contrast, no bacterium was detected in the blood of pups born from BM110ΔcylE-colonised mothers (Fig. 2j, k). The bacterial load was also quantified in the liver, an organ playing a key role during sepsis. As expected, pups born from BM110 WT-colonised mothers presented higher levels of GBS than those born from BM110ΔcylE-colonised mothers (Fig. 2l). GBS was also quantified in the brain of pups and although the blood of neonatal mice born from mothers colonised with the attenuated strain was negative for GBS, bacteria were detected in their brains up to PND15 (Fig. 2m). The level of brain colonisation was always slightly less than that observed in pups born from BM110 WT-colonised mothers, but the differences were not significant except at PND5 (Fig. 2m). The percentage of pups with brain colonisation at PND5 was 94% (15 out of 16) for those infected with BM110 and 50% (8 out of 16) for those infected with BM110ΔcylE (Fig. 2n). This indicates that almost all pups surviving the infection caused by the hyper-virulent strain have experienced meningitis.

To confirm that moribund animals infected with BM110 WT were dying from sepsis-related multiple organ failure, serum levels of several biochemical parameters were quantified at PND1 in pups born from BM110-colonised mothers (BM110) and in pups born from non-colonised mothers (Uninfected). The levels of aspartate transaminase [AST (U L^−1^), BM110: 685.0 ± 8.66 and Uninfected: 32.50 ± 12.99], and creatine kinase [CK (U L^−1^), BM110: 7625 ± 4021 and Uninfected: 60.00 ± 23.09] were much higher in the serum of pups infected with BM110 WT than in the serum of uninfected pups. These results confirmed liver and heart failure in the group of infected pups. No significant differences were observed in the serum levels of creatinine [CREA (µmol L^−1^), BM110: 2.50 ± 0.87 and Uninfected: 0.625 ± 0.22] and urea nitrogen [BUN (mmol L^−1^), BM110: 16.00 ± 0.577 and Uninfected: 17.50 ± 1.44] between the infected and uninfected groups indicating that the kidneys were functional.

The IAP administration of ampicillin or penicillin to GBS-colonised pregnant women is routinely used to prevent neonatal diseases. Therefore, BM110-colonised pregnant mice were prophylactically treated with ampicillin added to their drinking water from gestational day 20 (one day before delivery) until PND1. A significantly increased survival was observed in the group of pups born from IAP-treated mothers (87% survival), as compared to those born from untreated progenitors (59% survival) (Supplementary Fig. 2a). Moreover, at PND5, bacterial counts in the organs tested were below detection level in almost all pups born from IAP-treated mother, with only 1 out of 9 pups displaying gut and brain colonisation (Supplementary Fig. 2b–f). These results suggest that our mouse model can be used to test the efficacy of new prophylactic treatments against neonatal GBS infections.

### GBS-induced meningitis in neonates born from colonised mice

**Fig. 3:**
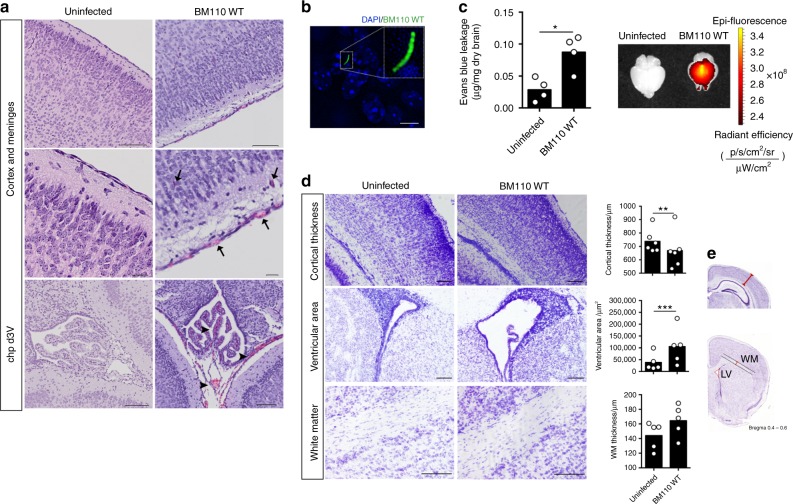
Prenatal-GBS infection leads to brain damage in the offspring. Pregnant BALB/c mice were intra-vaginally inoculated with 3 × 10^4^ CFU of GBS BM110 WT or PBS (uninfected) at gestational days 17 and 18. **a** Sections of brain tissue were prepared at PND5 and subjected to H&E staining. Representative micrographs of individual pups infected with GBS compared with uninfected control at indicated brain regions are shown. Arrows indicate oedema, vascular congestion and meningeal thickening in the cortex. Arrowheads depict vascular congestion and haemorrhage in the choroid plexus in the dorsal third ventricle. Chp d3v, choroid plexus dorsal third ventricle. Scale bars are 100 μm for top and bottom, and 20 μm for middle. **b** Immunofluorescence detection of GBS in the brain of an offspring born from a GBS-colonised progenitor at PND5. Scale bar, 5 μm. **c** BBB permeability measured by the quantitative analysis of Evans blue leakage to the CNS at PND5. Vascular permeability of the Evans blue dye is expressed as µg of Evans blue per mg of dried brain tissue using a standard curve. Representative fluorescence images of uninfected and infected pups’ brains analysed with the IVIS LUMINA LT are shown. Each dot represents data from a single pup (mean, *n* = 4 for uninfected and BM110 WT). Comparison by unpaired Student’s *t*-test. *P < 0.05. **d** Representative Nissl coronal sections showing cortical thickness, lateral ventricles enlargement and white matter (WM) thickness in pups infected with GBS BM110 WT compared with uninfected controls. **e** These modifications were quantified in brains of uninfected and infected pups. Measures were obtained as shown by red lines in representative sections (Image credit: Allen Institute). Scale bar, 100 μm. Each dot represents data from a single pup (mean, *n* = 5 for ventricular area and WM thickness, *n* = 6 for cortical thickness for both uninfected and BM110 WT). Comparisons by two-way ANOVA, with three replicate measurements per pup. **P < 0.01, ***P < 0.001

GBS-induced meningitis is often associated with long-term NDI and epidemiological studies have revealed that it is caused mainly by strains belonging to the hyper-virulent CC17 lineage^22–24^. Therefore, we compared at PND5 the brain of pups born from BM110-colonised mothers (infected pups) with those born from non-colonised progenitors (uninfected pups). The brain of infected pups exhibited the hallmarks of meningitis such as meningeal congestion, vascular hyperaemia, and oedema (Fig. 3a). Moreover, haemorrhage and hyperaemia were also found in the choroid plexus located in the dorsal third ventricle of infected pups (Fig. 3a). No histological alterations were observed in the brain of infected pups (Fig. 3a, c, e). Immunofluorescence analysis revealed the presence of GBS within the brain parenchyma (Fig. 3b). To investigate blood–brain barrier (BBB) breakdown, its integrity was evaluated in the pups’ brains at PND5 by the Evans blue (EB) dye assay. Higher values of vascular permeability were detected in the brains of infected pups, as compared to those observed in uninfected pups (Fig. 3c, left). The EB extravasation was further visualised by fluorescence imaging using the IVIS Lumina system (Fig. 3c, right). Cortical thickness, measured as indicated in Fig. 3e, was significantly decreased in brains from infected pups evidencing higher neuronal density and poorer discrimination of the different cortical layers (Fig. 3d). This may result from the observed expansion of lateral ventricles (Fig. 3d), a likely consequence of GBS ventriculitis^29^. However, the thickness of the white matter (corpus callosum) of brains from infected pups was not modified (Fig. 3e) and regions of abnormal necrotic death were not observed.

### Microglia activation and neuronal apoptosis in infected brain

**Fig. 4:**
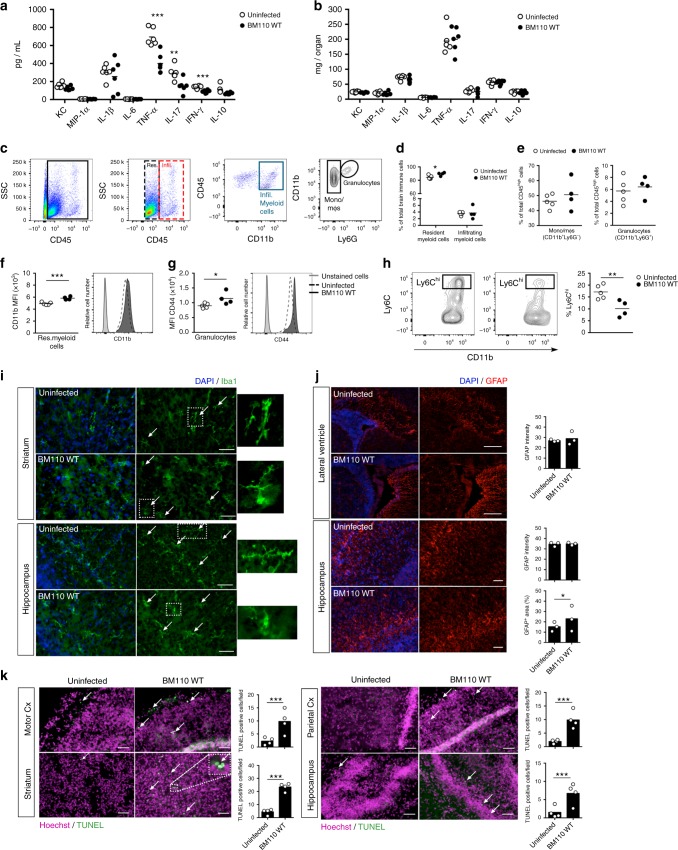
Prenatal-GBS infection induces microglia activation and neuronal apoptosis in the offspring. Pregnant BALB/c mice were intra-vaginally inoculated with 3 × 10^4^ CFU of GBS BM110 WT or PBS (uninfected) at gestational days 17 and 18. Analyses were performed at PND5. **a**, **b** Levels of serum (**a**) and brain (**b**) cytokines were quantified. Each dot represents data from one pup (mean, *n* = 6). Comparison by unpaired Student’s *t*-test. **P *<* 0.01, ***P < 0.001. **c** Representative flow cytometry plot scheme showing gating strategy. SSC: side scatter, Res: resident, Infil: infiltrating, Mono: monocytes, mϕs: macrophages. **d**, **e** Quantification of CD45^low/int^CD11b^+^ (resident myeloid cells), CD45^high^CD11b^+^ (infiltrating myeloid cells), CD11b^+^Ly6G^−^ (Mono/mϕs) and CD11b^+^Ly6G^+^ (Granulocytes) populations [mean, *n* = 4 (uninfected) and *n* = 5 (BM110 WT)]. Comparison by unpaired Student’s *t*-test. *P < 0.05. **f**, **g** Quantification of CD11b on microglia (**f**) and CD44 on granulocytes (**g**), presented as median fluorescence intensity (MFI). Representative histograms are shown. Black line, pups infected with BM110 WT; dotted line, uninfected pups; grey line, unstained cells. Mean, *n* = 4 (uninfected) and *n* = 5 (BM110 WT). Comparison by unpaired Student’s *t*-test. *P < 0.05, ***P < 0.001. **h** Quantification of Ly6C^hi^ on infiltrating Mono/mϕs with representative dotplots [mean, *n* = 4 (uninfected) and *n* = 5 (BM110 WT)]. Comparison by unpaired Student’s *t*-test. **P < 0.01. **i** Representative images of striatal and hippocampal microglia stained with Iba1. Arrows indicate relevant morphologies, as depicted in lateral panels of isolated cells. Nuclei were stained with DAPI. Scale bar, 50 µm. **j** GFAP-stained astrocytes in representative coronal sections of the lateral ventricles and hippocampus (CA3). GFAP intensity and area were quantified to evaluate astrocytes reactivity. Nuclei were stained with DAPI. Scale bar, 50 µm. Each dot represents data from a single pup (mean, *n* = 3 for uninfected and BM110 WT). Comparisons by two-way ANOVA with repeated measures. *P < 0.05. **k** Quantification and representative coronal sections, processed for TUNEL assay, of motor and associative cortical regions, striatum and hippocampus (CA3). Nuclei were stained with Hoechst. The arrow indicates TUNEL-positive nuclei showing DNA fragmentation and apoptosis. Scale bar, 50 µm. Each dot represents data from a single pup (mean, *n* = 4). Comparisons by two-way ANOVA with repeated measures. ***P < 0.001

To investigate whether brain inflammatory response was present, cytokines known to be relevant for bacterial meningitis was quantified in serum and brain of infected and uninfected pups at PND5. The levels of tumour necrosis factor (TNF)-α, interleukin (IL)−17 and interferon (IFN)-γ were significantly lower in the serum of infected pups compared to uninfected pups (Fig. 4a) whereas the levels of keratinocyte chemoattractant (KC), macrophage inflammatory proteins (MIP)−1α, IL-1β, IL-6 and IL-10 were similar (Fig. 4a). No differences were observed in the levels these 8 cytokines measured in brains of infected and uninfected pups (Fig. 4b). To gain insight into the mechanism responsible for brain damage, we next analysed the leucocytes present at PND5 in the brain of pups born from BM110-colonised mothers together with the status of microglia activation. In this analysis, to distinguish resident from infiltrating myeloid cells, we used a common classification method based on CD45 expression, which is higher on infiltrating cells^30^. Cells were gated on CD45^+^ immune cells and among CD45^low^ cells (resident cells) those expressing CD11b^+^ were identified as resident myeloid cells (commonly known as microglia), whereas the CD11b^+^CD45^hi^ cells were identified as infiltrating myeloid cells (Fig. 4c). We further gated CD45^hi^CD11b^+^ to distinguish between granulocytes (CD11b^+^Ly6G^+^) and monocytes/macrophages (CD11b^+^Ly6G^−^) (Fig. 4c). A significant increase in the frequency of resident myeloid cells was observed in brains from infected pups compared with brains from uninfected pups while no significant difference was detected in the infiltrating myeloid cells population (Fig. 4d, e). Higher expression of CD11b was observed in the resident myeloid population of brains from infected pups compared to those from uninfected pups (Fig. 4f). Increased expression of CD11b levels is associated with resident myeloid cells activation^31^, indicating that the microglia of infected pups was activated. No alterations were detected in the expression levels of MHC class II and Ly6C in resident myeloid cells (Supplementary Fig. 3a, b). Although no differences were observed in the levels of the CD11b^+^Ly6G^+^ population between brains from infected and uninfected pups, the mean fluorescence intensity of CD44 (an important receptor for cell motility upregulated upon cell activation) was significantly increased in brains from infected pups (Fig. 4g), indicating neutrophil activation. A significant decrease in Ly6C^hi^ inflammatory monocytes involved in brain homoeostasis^32^ was observed in brains from infected compared to those from uninfected pups (Fig. 4h). Concomitantly, the proportion of Ly6C^int/low^ monocytes was increased in brain from infected pups.

Since pathological stimuli affect both microglia functional state and anatomical structure^33^, we used Iba1^+^ staining to visualise changes in microglia morphology at PND5 in brains from infected and uninfected pups. At this time-point, microglia is usually uniform throughout the brain despite the presence of microglia region-specific phenotypes^34^. As expanded lateral ventricles were observed in brains from infected pups, we focused our attention on the striatum and hippocampus (CA3 region). In both regions (Fig. 4i), a shift towards an insult-responsive phenotype was observed in infected pups, characterised by reduced branching and increased soma size^33^, which is coherent with the observed activation state (Fig. 4f).

Astrocytes are important components of BBB and blood–cerebral spinal fluid (CSF) barrier and they respond to GBS infection by releasing several inflammatory cytokines, which may lead to reactive astrogliosis^35,36^. Therefore, inflammatory activation of astrocytes was evaluated at PND5 using GFAP staining in the lateral ventricles and in the hippocampus of brains from infected pups and brains from uninfected pups. Although the GFAP intensity was not different between the groups (Fig. 4j), the area occupied by GFAP^+^ positive cells in the hippocampus was larger in brains from infected than in brains from uninfected pups (Fig. 4j), indicating moderate reactive-astrogliosis. The periventricular area occupied by GFAP^+^ cells could not be compared between the groups due to ventricular expansion.

To evaluate whether apoptosis contributes to the neuropathology, neuronal apoptosis was assessed at PND5 by TUNEL nuclei in the hippocampus, striatum, motor and parietal cortical regions, and posteriorly at the parietal associative cortex of brains from infected and brains from uninfected pups. As illustrated in Fig. 4k, an increased apoptotic cell death was observed in all assessed regions of the brains from infected pups.

### Behavioural alterations in survivors of GBS meningitis

**Fig. 5:**
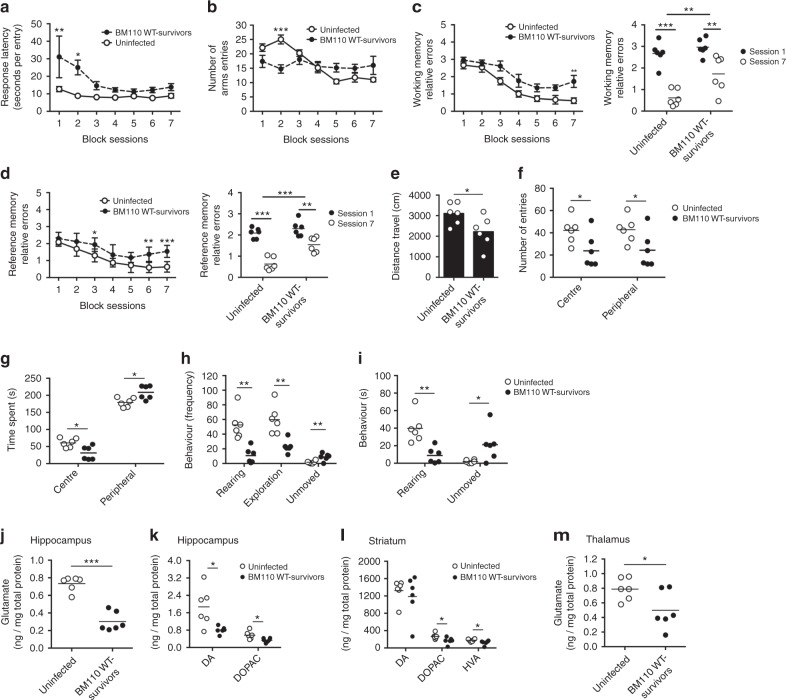
GBS-Survivors mice present behavioural sequelae and altered glutamatergic and dopaminergic function, in adulthood. Pregnant BALB/c mice were intra-vaginally inoculated with 3 × 10^4^ CFU of GBS BM110 WT or with PBS (uninfected) at gestational days 17 and 18. Offspring that survived to infection (BM110 WT-survivors) and mice born from non GBS-colonised mothers (uninfected) were examined at PND90 for their capacity to execute tasks in the radial arm maze (**a**–**d**) and tested in an open field (OF) test (**e**–**i**). **a**–**d** Latency (**a**), total number of arms entries (**b**), working memory errors (**c**) and reference memory errors (**d**) in the radial arm maze test. **e-i** Total distance travelled in centimetres (**e**). Frequency (**f**) and time spent in the periphery and centre of OF apparatus (**g**). Frequency (**h**) and time of exploratory behaviour (**i**). Data are presented as means ± SEM, or individually (*n* = 6 for uninfected and BM110 WT-survivors). Comparisons by two-way ANOVA, with repeated measures for block sessions or unpaired Student’s *t*-test for the other evaluations. *P < 0.05, **P < 0.01, ***P < 0.001. **i-m** Levels of Glutamate in the hippocampus (**j**) and thalamus (**m**), and DA and metabolites (DOPAC and HVA) in the hippocampus (**k**) and striatum (**l**) of offsprings that survived to neonatal GBS infection (BM110 WT-survivors) or born from non GBS-colonised mothers (uninfected), were determined by HPLC-EC at PND90. HVA levels in the hippocampus were below the detection limit. Relative levels are shown and normalised to total protein content. Each dot represents data from a single animal (mean, *n* = 6 for uninfected and BM110 WT-survivors). Comparison by unpaired Student’s *t*-test. *P < 0.05, ***P < 0.001

To assess if, in our model, the offspring that survived infection with the hyper-virulent strain experienced permanent NDI, as observed in humans, we characterised their cognitive and motor performance in adults. The learning and memory performances were assessed using an 8-arm radial maze task^37^. A significant difference between GBS-Survivors (BM110-Survivors) and mice born from non-colonised mother, control group (Uninfected), was observed regarding the latency to enter to the first arm in the first two block sessions (Fig. 5a), where the GBS-Survivors took significantly more time to start exploring the maze. While the Uninfected group presented a classical learning curve, with a number of arm entries decreasing as time progressed, the GBS-Survivors group did not alter the number of entries throughout the test (Fig. 5b). This is further evidenced in working and reference memory performances, for which GBS-Survivors present higher relative error rates at the final sessions, indicating a diminished ability to learn the task (Fig. 5c, d). General motor function and exploratory behaviour were assessed in the open field test (OFT). GBS-Survivors display a significant decrease in the total distance travelled in the OFT (Fig. 5e). Importantly, both the number of crosses between the peripheral and central area and the time spent in the central area were significantly reduced for the GBS-Survivors group (Fig. 5f, g), which is a sign of anxious-like behaviour. When analysing the exploratory activity of GBS-Survivors, data revealed that these animals present a significantly lower frequency and time spent in investigatory behaviours such as rearing and exploration, when compared to controls (Fig. 5h, i). These animals also showed an increase of immobility time and frequency (Fig. 5h, i). Globally these results show that GBS-Survivors presented compromised activity and exploration abilities, as well as anxiety-related behaviours.

### GBS meningitis leads to an altered neurotransmitter pattern

To understand if the observed learning and behaviour changes in GBS-Survivors mice are related to the levels of relevant neurotransmitters (dopamine and metabolites, norepinephrine, glutamate, and GABA), these molecules were quantified in brain regions involved in memory acquisition, learning and motor activity by HPLC-ED.

In the hippocampus, a major glutamatergic region that receives relevant dopaminergic input, we observed significantly reduced levels of glutamate, dopamine and its metabolite DOPAC for the GBS-Survivors (BM110-Survivors) (Fig. 5j, k). In the striatum, a predominantly GABAergic region modulated by nigral dopaminergic inputs and cortical glutamatergic projections, we observed reduced levels of dopamine metabolites (DOPAC and HVA) for the GBS-Survivors group (Fig. 5l), which are indicative of decreased dopaminergic turnover. Altered dopaminergic activity in this region is associated with thalamic deregulation of glutamate release^38^. Accordingly, we observed in the thalamus a significant reduction of glutamate levels for the GBS-Survivors group (Fig. 5m), which is also consistent with a decreased activity. No other differences were observed in the levels of dopamine, norepinephrine, glutamate or GABA in any of the evaluated brain regions (hippocampus, striatum, prefrontal cortex, amygdala, and cerebellum) (see Supplementary Tables 1 and 2).

Globally, mice born from BM110-colonised mothers present a meaningful number of alterations in learning ability as well as in the spontaneous behaviour, which is in agreement with the observed decreases in glutamatergic and dopaminergic functions in the hippocampus and the striatal-thalamic circuit.

## Discussion

We here describe a novel mouse model that reproduces all steps of GBS infection in humans. Transmission of this bacterium from mother to the neonate results either from ascending spread of bacteria or, alternatively, through ingestion or aspiration of contaminated vaginal fluids during delivery^2^. Maternal vaginal colonisation is thus the critical initial step of this invasive neonatal disease. To validate this model, we used the GBS strain BM110 belonging to the hypervirulent lineage CC17 responsible for approximately 80% of meningitis cases due to GBS in neonates and the attenuated mutant derivative BM110∆cylE not expressing the β-hemolysin/cytolysin. BM110 efficiently colonised the vaginal mucosa of pregnant females and a transient and intermittent vaginal colonisation was observed after delivery, as observed in humans^39^. Most importantly, the hypervirulent strain colonises the vagina of pregnant mice more efficiently than the attenuated strain. These results confirm previews studies showing that β-hemolysin/cytolysin expression is involved in adherence of GBS to the murine vaginal tract of nonpregnant mice following treatment with 17β-estradiol^40,41^.

In human neonates, the lung is the primary organ infected by GBS upon vaginal fluids aspiration, with one-third to more than half of infants showing lung symptoms within hours after birth^2^. In our murine model, 60% of pups infected with the hyper-virulent GBS strain that survived (a percentage similar to that reported in humans before IAP introduction) presented respiratory pathology. In addition, these pups had elevated GBS levels in their lungs later in life, which is consistent with previous studies reporting that throat and rectal cultures are the best sites for GBS detection in childhood^42,43^. As expected, pups infected with the β-h/c toxin-deficient strain had low levels of systemic colonisation. However, both strains were detected in the gut during the neonatal period and adulthood in animals, revealing that GBS-commensalism is likely established during early life, without the contribution of β-h/c. These data support the hypothesis that the gastrointestinal tract is the GBS reservoir and the most likely source of vaginal colonisation in humans^44,45^. Whether this form of commensalism is a cause of transition to invasive niches remains unknown. As reported in an adult murine model of haematogenous GBS meningitis^25^, bacteria were found in the brains of neonatal mice infected with the WT or the β-h/c-deficient mutant strain, albeit at lower levels with the mutant strain. Consistently, screening of a large collection of human GBS isolates led to the suggestion that β-h/c production contributes to, but is not essential for virulence, as non-haemolytic isolates were found in strains originating from invasive diseases including CC17 strains^46^. Therefore, more exploration is needed to understand the role of non-hypervirulent GBS infections in brain damage and behavioural sequelae.

Previous mouse models of intra-vaginal infection during pregnancy have also been described^26,47^. However, to follow ascending GBS infection to the foetus and preterm birth, a protocol with a high bacterial inoculum was used, leading to infection and severe intrauterine foetal demise with significant maternal bacteraemia^26,47^. Our model is different because newborns acquire the bacterium during delivery mainly by ingestion or aspiration of vaginal secretions colonised by GBS, which allows substantial neonatal survival and thus the study of meningitis pathophysiology. Moreover, we confirmed that pregnant females inoculated with a hypervirulent mutant strain CC17 lacking β-h/c showed a decrease in vaginal colonisation and vertical transmission to offspring, as compared to the haemolytic parental strain^26^.

A minimum bacteraemia threshold is considered required for meningeal invasion^48^. However, we show that at PND1, 40% of infected animals had bacteraemia below the detection threshold, and this percentage decreases to 25% at PND5 although ~94% of pups had bacteria in their brain. This observation is very important as human blood cultures are negative in 15–38% of cases^12^. It should be noted that 50% of pups infected with the mutant strain β-h/c also had GBS in their brain with no bacteria detected in the blood. Thus, our model summarises the pathogenesis of human GBS infection and is able to discriminate the effects of the hyper-virulent strain and an attenuated mutant derivative. Hence, it is different from a model of GBS-induced maternal infection leading to a deleterious neurodevelopmental impact on uninfected offsprings, and which does not discriminate between inactivated and live GBS^49^. However, our model does not allow the study of premature births and foetal deaths caused by GBS and, because of the size of the animal, the collection of CSF samples. Moreover, it does not clearly distinguish between the early-onset and late-onset diseases. Lastly, it does not allow investigating the possible transmission of GBS via infected breast milk^50^ or hematogenous and peritoneal routes^51^.

Although meningitis is classically defined as an inflammatory disease of the meninges, it is not limited to these membranes, and adverse consequences on brain dysfunction seem to be, at least partially, associated with the host neuroinflammatory response^52^. Glia activation is one of the first hallmarks of neuroinflammation^53^, but the specific contribution of different cell types to brain inflammation and cell death in GBS-induced meningitis remains unknown. The initial inflammatory response is thought to depend on activation of innate immune receptors^54^. Direct RNA sequencing analysis demonstrated that microglia express high levels of several sensome genes, including the Toll-like receptor 2 (TLR2)^55^ and this receptor is up-regulated in the neonatal brain under physiological conditions^56^. Moreover, TLR2-signalling might has detrimental effects in the immature brain^54^, and during neonatal sepsis^57^. Peripheral administration of the TLR2 ligand Pam3CSK4 to neonatal mice induced a dramatic accumulation of neutrophils and monocytes in the CSF, indicating that systemic activation of TLR2 leads to an increased inflammatory response in the brain^58^. Here, we did not observe the recruitment of leucocytes into the brain at a time when systemic infection persists. The absence of neutrophil infiltration was also reported in a neonatal stroke model^59^. However, we observed a decrease in the proportion of Ly6G^-^Ly6C^hi^ monocytes and a smaller increase in Ly6G^-^Ly6C^int/low^ monocytes in the brains of infected pups. Ly6C^hi^ monocytes deficiency has been associated with altered hippocampal neurogenesis^32^ and Ly6G^-^Ly6C^in/low^ monocytes have recently been associated with impaired motor learning and dendritic spinal plasticity related to learning^60^.

Microglia undergoes rapid activation and proliferation upon brain injury. Accordingly, we found increased microglia activation and a morphology shift towards a reactive phenotype^33^ that was particularly evident in the dorsal striatum, near the lateral ventricles in infected brains where apoptosis was also more expressive.

Apoptosis and reactive astrogliosis, another hallmark of neuroinflammation, were observed in the hippocampus of infected pups. This brain region is frequently associated with cognitive neuronal sequelae induced by GBS meningitis^61^. Expansion of the lateral ventricles was also observed in the brain of infected pups. This is a likely consequence of the on-going ventricular neuroinflammation that may evolve to ventriculitis and hydrocephalus, two possible complications of GBS-induced meningitis^29^. It should be noted that the cortical thinning observed in the brains of infected pups, likely resulting from an expansion of lateral ventricles^29^, may impair cognitive function at later stages. We observed a lower concentration of some inflammatory cytokines in the serum of infected pups that could be explained by the unique features of the neonatal immune system highly regulated by multiple factors^62^.

No differences were observed for the levels of inflammatory cytokines in the brain of infected or uninfected pups. Thus, brain damage is unlikely due to local or systemic inflammatory cytokines. Moreover, it is also unlikely that brain cells are directly damaged by bacteria, as apoptosis was observed in several regions with no detectable GBS. Therefore, neurotoxicity molecules such as reactive oxygen species, nitric oxide, peroxynitrite, metalloproteinases, and excitatory amino acids, can be the responsible for neuronal injury associated with GBS meningitis^63,64^. Altogether, these data support the hypothesis that local, rather than systemic inflammation, contributes to NDI in GBS meningitis.

Though data are scarce concerning permanent NDI in surviving neonates, a study showed that, despite reduced mortality (5.5%), long-term outcomes of GBS meningitis were remarkably similar to a report from 1985^9,11^. These studies showed that among the survivors of an initial episode of GBS meningitis, one-half presented global or mild-to-moderate mental retardation, including learning disabilities, and language deficits, with associated neurologic abnormalities. More recently, a systematic literature review and meta-analysis for NDI outcomes due to GBS meningitis showed that almost one-fifth of survivors presented moderate to severe NDI^10^. It is likely that this value is underestimated due to limited data from low and middle-income populations, which accounts for more than 90% of the world’s births and where health care systems are often poorly developed^10^. In our experimental model, the GBS-Survivors mice presented reduced exploratory activity in adulthood as well as impairment in learning and memory as evaluated in the radial maze. The reduced activity observed did not seem to affect the learning process since the number of increased errors observed in GBS-Survivors performance is not globally a reflex of reduced arms entries. Altered neurotransmitter patterns in several brain regions further sustained these differences. Glutamate, that plays a crucial role in learning and memory formation^65^, was decreased in the hippocampus of GBS-Survivors mice and could reflect the learning and memory disabilities found in children that survive meningitis^66^. The dopaminergic system is usually associated with the control of movement and decision making^67,68^. Dopaminergic inputs into the hippocampus and the frontal cortex are also relevant to learning and attention^67^. In our model, the levels of DA and its metabolite DOPAC were significantly decreased in the hippocampus from GBS-Survivors, justifying the impairments observed. The basal ganglia are involved in reward-based learning and motor function^68^. The major site of synaptic plasticity in the basal ganglia is the striatum, which receives cortical glutamatergic inputs and dense dopaminergic projections^69^. Mice that survived to neonatal GBS infection displayed reduced DA metabolism in the striatum, which is in agreement with reduced global activity, and a likely consequence of the postnatal inflammatory response described above. Of note, this can lead to decreased influx of glutamate from the thalamus to the motor cortex and result in hypokinesia^70^. In accordance, we detected significantly lower glutamate levels in the thalamus from GBS-survivor animals, which may also impact on other areas of the neocortex, since glutamatergic afferents from the thalamic region serve as a cortex relay^71^.

**Fig. 6:**
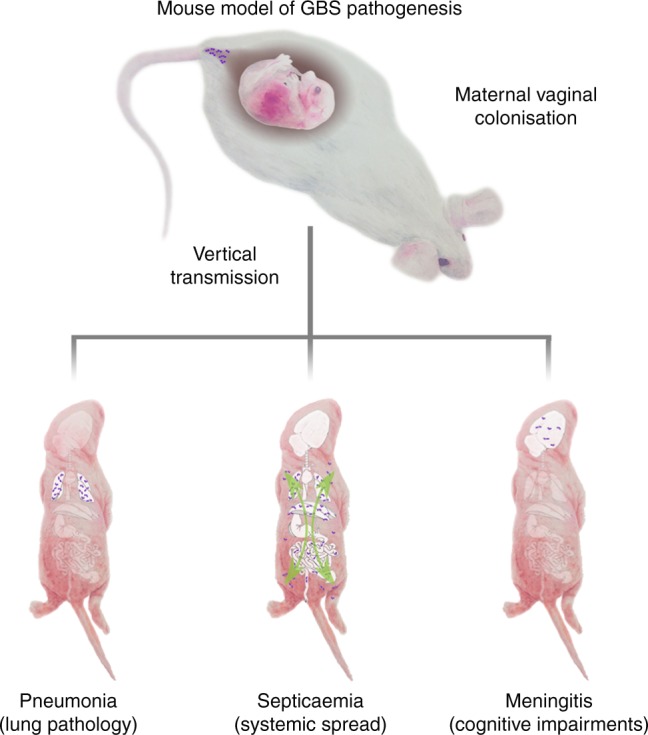
Neonatal mouse model of GBS pathogenesis. GBS colonises the vaginal tract of pregnant mice. The newborn acquires the bacteria during birth, causing pneumonia, septicaemia, and meningitis. Scientific illustration by Gil Ferreira da Silva

Our experimental model closely mimics the human GBS infection during birth and is the first that enables a mother-to-child transmission leading to GBS-induced diseases (Fig. 6). It constitutes a useful tool to study the pathophysiological mechanisms of CNS inflammatory response during GBS meningitis, as well as to study how bacteria circumvent the blood–CNS barriers, aiming at reducing neurological and neuropsychiatric morbidities in maturing newborns.

## Methods

### Bacterial strains and growth conditions

GBS strain BM110 (here referred to as BM110 WT), a capsular serotype III strain belonging to the hyper-virulent clonal complex 17 (CC17), is a well-characterised isolate from humans with invasive infections^72^. The isogenic non-haemolytic (βH/C-deficient) Δ*cylE* mutant (here referred to as BM110ΔcylE) was constructed by deleting in-frame the gene *cylE* as previously described^73^. GBS BM110 WT and the ΔcylE mutant were cultured at 37 °C in Todd-Hewitt broth or agar (Difco Laboratories) containing 5 µg/mL of colistin sulphate and 0.5 μg/mL of oxalinic acid (Streptococcus Selective Supplement, Oxoid).

### Animals and ethics statement

Six- to eight-week-old male and female BALB/c mice were purchased from Charles River. All animals were kept at the ICBAS animal facilities during the time of the experiments. All experiments were conducted in strict accordance with the recommendations of the European Convention for the Protection of Vertebrate Animals used for Experimental and Other Scientific Purposes (ETS 123) and Directive 2010/63/EU and Portuguese rules (DL 113/2013). All experimental protocols including animals were approved by the competent national authority Direcção Geral de Alimentação e Veterinária (DGAV), and by the ICBAS Animal Ethical Committee (No 113/2015). People directly involved in animal experiments were also certified by DGAV. All efforts were made to minimise animal suffering and to reduce the number of animals used. No animals were excluded from the analysis. Since our experiments were designed to colonise progenitors during pregnancy, we did not randomise their litters. No blinding was carried out.

### Gestation time and pregnancy monitoring

Since the presence of vaginal plug is not a definitive indicator of true pregnancy, particularly in inbred mice^74^ body weight was used jointly to determine the time of gestation. Two to three females were housed with one male and examined daily for the presence of vaginal plug at the beginning of the light cycle. When the vaginal plug was observed, the female was separated and housed individually until delivery. Nest material was provided to each dam and no bedding changes were performed during the last days of pregnancy. The day of the vaginal plug detection was considered as gestation (G) day one (G1) and the day of delivery designated as postnatal day (PND) 0. All animals were accustomed to the investigator, in order to reduce stress. Pregnant BALB/c mice were intra-vaginally (i.vag.) inoculated at G17 and G18.

### Neonatal mouse model of GBS meningitis

Overnight GBS cultures were subcultured 1:100, grown until mid-log phase (OD_600_ ~0.800), pellet and washed twice with sterile phosphate-buffered saline (PBS). Pregnant BALB/c mice were i.vag. inoculated with 40 µL containing 3 × 10^4^ GBS cells using a micropipette. This period of intervention to bacterial administration has been determined to be the optimal, as early administration did not allow pregnancy to reach term (data not shown). Pregnant females were allowed to deliver by spontaneous partum that occurred at G20-G21 and infected pups were kept with their mothers during the first 30 days after birth. When indicated, a group of females received ampicillin (Sigma) in their drinking water (1 mg/mL), from 1 day prior to delivery (G20) until 1 day after birth. Vaginal lavages were collected by vaginally instilling 50 μL of sterile PBS with a micropipette and repeatedly (10 times) removing and introducing the PBS. This was repeated twice and 150 µL of vaginal lavage were recovered at the indicated time points. Vaginal lavages were serially diluted, plated on selective medium (CHROMagar StrepB agar) and incubated overnight at 37 °C. The litter size and pup weight were assessed during the first 10 days after birth. Survival curves were determined in a 30-day period monitored every day. To assess bacterial colonisation, the liver, lungs, gut and brain were aseptically removed at the indicated PND and homogenised in PBS. Serial dilutions were prepared in sterile saline, and plated for CFU counts. Blood was collected by decapitation to avoid extreme hypovolaemia associated with stress and pain. Samples were collected in heparinized containers and plated for CFU counts. At the indicated time points, gut was collected and recovered GBS were enumerated by light pink or mauve colonies on CHROMagar StrepB agar.

### Blood–brain barrier permeability to Evan’s blue

The blood–brain barrier integrity was investigated using Evan’s blue dye extravasation. At PND5 neonatal mice were intraperitoneally injected with a 2% solution of Evans Blue in PBS (4 µl/g of body weight). Three hours (h) later, animals were deeply anaesthetised and perfused with saline. The whole brain was removed and wrapped in aluminium foil. The organs were dried for 48 h in an oven at 55 °C and Evans Blue was extracted by addition of formamide (8 µl / mg brain). After an additional 48 h period at 55 °C, Evans Blue stain was measured by spectrophotometer at 620 nm and quantified according to a standard curve. The results were presented as µg of Evans Blue per mg of dried tissue. To visualise the fluorescence of Evans blue brains were following perfusion and fixation with paraformaldehyde (PFA), acquired with an IVIS Lumina LT (Perkin Elmer). The detection of the fluorescent signal by the system resulted in the generation of signal maps automatically superimposed to the grey-scale photograph of the brain using the Living Image software (Perkin Elmer).

### Blood biomarkers of organ injury

Serum samples from moribund BM110 WT-infected animals, or uninfected controls were collected at PND1. The following biochemical parameters were measured in the serum as markers of multiple organ failure: liver injury was assessed by measuring serum concentrations of aspartate transaminase (AST); kidney dysfunction was evaluated by measuring the increase in creatinine and blood urea nitrogen (BUN); and cardiac lesions were assessed by measuring creatine kinase (CK). The determinations were made using a commercial kit (Idexx laboratories).

### Histopathology examination and immunohistochemistry

For histopathology examination, brain and lungs were removed at the indicated time-points, fixed in 4% buffered formalin, routinely processed, and embedded in paraffin. Histologic 4–5 µm-thick sections were cut for staining or immune detection. Hematoxylin and eosin (H&E) staining were performed according to per standard protocols. H&E-stained sections were analysed by a pathologist (A.F.). Slides were assigned random numbers that had no relevance to the experiment for a blind analysis.

For immunohistochemistry analysis, animals were deeply anaesthetised, perfused with PBS, followed by 4% PFA in PBS. The brains were removed, post fixed by immersion in 4% PFA for 48 h, at 4 °C, washed with PBS and then cryoprotected using sucrose 30%. Samples were thereafter mounted in OCT (Thermo Scientific) embedding medium, frozen and cryosectioned in the CM3050S Cryostat (Leica Biosystems). Brain coronal sections (30 μm) were collected in Superfrost ultra plus slides (Thermo Scientific), and stored at −20 °C until processed. Immunohistochemistry analysis was performed as previously described, with slight modifications^75^. Briefly, glass slides containing frozen sections were defrost at least 2 h and then hydrated with PBS for 15 min. Sections were fixated with 4% PFA, washed three times, permeabilized with 0.25% Triton X-100 for 10 min and washed with PBS for 5 min. Sections were then blocked (5% BSA, 5% FBS, 0.1% Triton X-100) for 1 h and primary antibodies were incubated overnight in blocking solution in a humidified chamber overnight at 4 °C. The following antibodies were used: 1:500 anti-Iba-1 (Wako), 1:500 anti-GFAP (Abcam), 1:200 anti-Streptococcus Group B antibody (Abcam). Next, sections were washed three times for 10 min with PBS and relevant secondary antibodies [1:1000 Anti-Rabbit Alexa 488 (Cell Signalling Technology), 1:1000 Anti-Rabbit Alexa 594 (Thermo Fisher Scientific) and 1:400 Anti-Rabbit Alexa 568 (Thermo Fisher Scientific)] were incubated for 2 h in blocking solution. For nuclei visualisation, sections were washed three times for 10 min with PBS and incubated for 10 min with Hoechst or 4,6-diamidino-2-phenylindole (DAPI). The apoptosis was measured by TUNEL assay following instructions provided by the manufacturer (Promega). Slides were cover slipped using Fluoroshield (Sigma) and visualised under a Zeiss Axio Imager Z1 microscope. To assess cytoarchitecture sections were dipped in Cresyl Violet solution (0.5% cresyl violet in 0.3% acetic acid), rapidly dehydrated and mounted with DPX (BDH).

### Image analysis and quantification

For each animal, 3 images per region were obtained by selecting brain sections that where within Bregma 0.4 to 0.6 (striatal region) and −3.80 to −4.30 (hippocampus) as illustrated in Fig. 3e. Images were acquired using uniform settings and exported preserving original metadata. For Iba1 and GFAP images, fluorescence channels were separated, images converted to 32-bit and the background subtracted using 50%-off pixels radius by the rollerball algorithm. The amount of Iba1 positive pixels per total area was considered. For GFAP, deconvolved images were segmented in FIJI using an automatic local Otsu threshold algorithm (based sole on the maximum intensity in the distribution histogram). Mean grey values for the intensity and total area of GFAP expression were returned for each image using the measure function on Image J (version 1.50i for Mac). TUNEL positive cell were count manually using also 3 replicates per region and subject. Individual values for each image were exported to the Prism software (Prism 7 for Mac OS X) for statistical analysis.

### Flow cytometry of leucocytes in the brain on neonatal mice

For flow cytometry of brain tissue, pups at PND5 were deeply anaesthetised, perfused with saline, and euthanized by decapitation. Brains were excised, gently dissociated and cells were passed through a 100 µm cell strainer. Stock isotonic Percoll (GE Healthcare) (SIP) 90% (in HBSS without Ca^2+^ and Mg^2+^) was added to each cell suspension to obtain a final 30% SIP. Slowly the cell suspension was added on top of the 70% SIP avoiding mixing of the 70 and 30% solutions. Percoll gradients were centrifuged (500*g*, 30 min, at 22 ˚C) and the enriched population of leucocytes/microglia was collected at the 70–30% interphase. After isolation, cells were washed and counted in a CountessTM Automated Cell Counter (Thermo Fisher Scientific). Thereafter, 5 × 10^5^ cells were incubated with Ly6G FITC (Clone 1A8), CD45 PE (Clone 30-F11), Ly6C PerCP/Cy5.5 (Clone HK1.4), CD11b PE/Cy7 (Clone M1/70), MHC-II BV421 (Clone M5/114.15.2), and CD44 BV510 (Clone IM7), all from Biolegend, diluted in FACS staining buffer (2% BSA, 0,1% sodium azide in PBS). Data were acquired in a FACSCanto II flow cytometer (BD Biosciences). Post-acquisition analysis was performed using FlowJo software v10 (Tree Star). Gating strategy is shown in Fig. 4.

### Multiplex cytokine assay

Serum samples were collected at PND5. For brain analysis, animals were deeply anaesthetised and perfused with saline. Brain tissue samples were frozen on dry ice immediately upon removal. Brains were homogenised on ice in RIPA buffer (Sigma-Aldrich) containing protease inhibitor cocktail (Roche). The ground tissue was frozen at −80 °C and thawed to improve cell lysis. Lysates were pelleted by centrifugation (4500*g*, 4 min, 4 °C) and supernatants stored at −80 °C until use. Cytokine assay was performed by using a Bio-Plex custom 8-Plex kit (Bio-Rad) following the manufacturer protocol, and a Luminex MAGPIX Instrument. Serum samples were diluted 1:4 in diluent buffer, and brain suspensions were diluted 1:2 to load approximately 1 mg of protein. Detection limits were as follows: KC, 3.08 pg mL^−1^; MIP-1α, 1.26 pg mL^−1^; IL-1β, 1.14 pg mL^−1^; IL-6, 1.36 pg mL^−1^; TNF-α, 6.36 pg mL^−1^; IL-17, 1.1 pg mL^−1^; IFN-γ, 1.96 pg mL^−1^; IL-10, 9.24 pg mL^−1^. Data from brain were normalised relative to protein concentration.

### Behavioural assessments

The behavioural tests were performed in a sound attenuated, temperature (21 ± 1 °C) and humidity (70%) controlled room with a 12-h-light–dark cycle (lights at 7 a.m.), using male animals at PND90 (mature adults), from different litters. Females were not used due to concerns about hormones fluctuation associated with oestrous cycle, which produce greater variability in behavioural data^76^. In the radial maze test animals were kept at approximately 90% (26.64 ± 1.28 g) of their free feeding body weight and began training after reaching this weight. Since food restriction is required to induce motivation to search for food (pellet reward), during the whole test period mice had restricted access to food (only fed following testing). Their weight and general health were carefully monitored every day to prevent more than a 10% body weight loss.

Radial maze test: The cognitive status of the animals as regards spatial learning and memory was assessed with the 8-arm radial maze. Working and reference memory were assessed simultaneously through a fixed position reward task, in which half of the arms were baited and their positions were fixed throughout the training trails. The radial maze consists in a central area (22 cm in diameter) giving access to eight equally-sized arms in transparent acrylic (length, 25 cm; width, 6.5 cm). Identical food wells (2.5 cm deep and 3 cm in diameter) were placed at the distal end of each arm. Commercialised sugar pellets (Bioserv F0042 - DPP’S 45MG SUGAR 50TH) located at the end of each baited arm were used as rewards. Two days prior to the beginning of the test, the rewards were placed once a day in the animal cages, to allow them to explore and eat the pellets. There were extra-maze clues including free-standing laboratory equipment, and geometric pictures in walls to help mice navigation. A digital stopwatch was used to record the amount of time taken to a mouse to complete a trial.

Habituation/training: On the first habituation day, each animal was placed alone in the centre of the starting platform and allowed to freely explore the maze. On the second habituation day, the mice were allowed to explore the apparatus with randomly placed food pellets throughout the maze, for a 5 min period. On the third habituation day, the food pellets were placed at the distal end of each arm and the animals were allowed to explore for 5 min. The habituation period ended after the mouse ate at least 4 rewards or when 5 min had elapsed whichever came first.

Test: During the test phase, 4 out of 8 arms were baited and randomly assigned for each mouse, but always constant for the same animal throughout the trial period, and the mice were given a maximum of 5 min to complete the maze. Geometrical figures placed on the walls and the researcher were the only visual extra maze cues present. In each training session, the mouse was placed within a transparent cylinder on the platform in the middle of the maze for few seconds. The cylinder was then lifted and the animal was allowed to move freely in the maze. It was considered that an animal had entered an arm when the 4 paws and the tail were inside it. Trials ended when the animal had either eaten all 4 rewards or 5 min had passed, whichever came first. Once the animal returned to the central platform and the cylinder was lowered, a minute later the next trial took place. One session of two trials was performed per day over 14 days. The maze was rotated and wiped with a dextran solution at 2% between animals to eliminate or reduce olfactory cues from different individuals.

Behavioural analysis: There were 4 measures used in behavioural analysis. The number of errors made: working memory error, defined as re-entering an arm already visited within a trial; and a reference memory error, defined as entering a never baited arm. Trial completion time was also included. It started when the cylinder was lifted, in which the mouse had full access to explore the maze, and stopped when the animal entered the fourth baited arm. Response latency was also measured and defined by the total session duration divided by the number of arms entered (seconds per entry). Seven blocks were calculated by average measure of four trials per day.

Open field (OF): Exploratory behaviour and general locomotor function were assessed in the open field test. The apparatus consisted of a brightly illuminated open-field square arena (40 × 40 × 40 cm). The OF arena is virtually divided into 16 equal squares, via a 4 × 4 grid to assist with data analysis. In each session, mice were individually placed in the centre of the arena and allowed to freely explore it for 5 min. All trials were recorded by a video camera (SONY DCR-SF 290), suspended above the test arena, and analysed afterwards using the software package Observer XT 7.0 (Noldus Information Technology). The following parameters were assessed: total distance walked, frequency and time spent in centre or periphery section of the OF. The number of rears (i.e. mice reared on its hind paws, both on or off the walls), arena exploration and time spent unmoved were also scored. The apparatus was cleaned between subjects with a solution of 2% dextran.

### Neurotransmitter determination

After the completion of behavioural studies, mice were euthanized by decapitation and the brains rapidly microdissected on ice and quickly frozen at −80 °C. Amino acid levels and the levels of monoamines and their metabolites were measured by high performance liquid chromatography, combined with electrochemical detection (HPLC/EC), using a Gilson instrument (Gilson, Inc., Middleton, WI, USA), as previously described^77^.

Amino acids quantification: For gamma-aminobutyric acid (GABA) and glutamate evaluation, the prefrontal cortex, hippocampus, striatum and cerebellum were used. The day before the neurochemical determination, tissues were thawed, and homogenised in 200 µL of ice-cold 150 mM potassium buffer with phosphoric acid, through ultrasonication (Sonifier W-250, Branson Ultrasonics) and centrifuged at 16,000*g*, for 10 min at 4 °C. The supernatant was collected, diluted 1:2 in dilution solution (0.4 N perchloric acid, 0.4 mM sodiumm dissulfite, 0.9 mM EDTA), filtered thought a 0.2 µm nylon microfilter (Corning) at 16,100*g* for 5 min, at 4 °C, and stored at −20 °C overnight. Afterwards, samples were derivatized by adding 100 μl of NaOH 0.1 N and 15 μl OPA (10 mg mL^−1^ of *o*-phthaldialdehyde (OPA), 45.4 M sodium sulphite, 4.5% absolute ethanol in 327 mM borate buffer at pH 10.4) to 50 μL of sample. Samples were allowed to react at room temperature in the dark, for 10 min and then injected into the HPLC system. The mobile phase consisted of 0.06 M sodium dihydrogen phosphate, 0.06 mM EDTA and 20% methanol, pH adjusted to 4.4 with phosphoric acid and it was filtered and degassed. The flow was maintained at 0.8 mL min^−1^. Concentrations of GABA and glutamate were calculated using a standard curve generated with a glutamate and GABA standard (Sigma-Aldrich). Final results were expressed in terms of amino acid content per total amount of protein.

Monoamines and their metabolites quantification: Levels of norepinephrine (NE), dopamine (DA), 3,4-diydroxyphenylacetic acid (DOPAC) and homovanillic acid (HVA) were determined in the striatum, prefrontal cortex, hippocampus and amygdala. The different brain regions were ultrasonicated in 200 µL of ice-cold 0.2 M perchloric acid and centrifuged at 13,000*g* for 3 min at 4 °C. The supernatant was then collected and filtered as described above. Aliquots of 120 µL were injected into the HPLC system, using a mobile phase of 70 mM of potassium phosphate monobasic buffer (pH adjusted to 3.0 by adding phosphoric acid) in 10% (v/v) of methanol, 1 mM 1-heptanosulfonic acid and 107.5 mM Na-EDTA. The flow was maintained at 0.8 mL min^−1^. Concentrations of neurotransmitters were calculated using standard curves generated with standard monoamines (Sigma). Final results were expressed in terms of monoamine content per total amount of protein.

### Total protein determination

Protein content was determined using the BCA Protein Assay Kit (Thermo Scientific-Pierce). Briefly, bovine serum albumin (BSA) was used as a standard protein (0.01–0.5 mg mL^−1^). 10 µL of sample or BSA standard in duplicate were loaded into a microplate and added 200 µL of working reagent to each well. After 30 min incubation at 37 °C, absorbance was read at 562 nm using a microplate reader.

### Statistical analysis

All data were analysed with the GraphPad Prism software (v.7, GraphPad software Inc. CA). Means and standard errors of the means (SEM) were calculated and correspond to the indicated independent experiments. The log-rank (Mantel–Cox) test was used to analyse the survival curve. The data from radial maze test were submitted to analyses of variance (ANOVA) with repeated measures for block sessions. Each block-session was calculated by average measure on two successive trials. Differences between multiple groups were analysed by one-way or two-way ANOVA, whenever appropriate, with the Sidak’s Multiple Comparison Test, for *α* = 0.05. Image analysis was made using two-way ANOVA with repeated measures. All other comparisons were done with an unpaired student’s *t* test. Normality was verified by the Shapiro–Wilk normality test. Homogeneity of variance was estimated by an *F* test. Assumptions of sphericity in ANOVA were also verified. The minimum sample size was determined using G*Power analysis for two-tail *t*-test comparing the mean and standard deviation of two independent groups with effect size *d* = 7, *α* = 0.05 and power of 0.95. CFU data were log10 transformed. Significance was represented by the following symbols: *P < 0.05, **P < 0.01, ***P < 0.001, ****P < 0.0001, and n.s. not significant.

### Data availability

All relevant data supporting the findings of the study are available in this article and its Supplementary Information files, or from the corresponding author upon request.

## Electronic supplementary material

Supplementary Information
