# Supplementary material for: A mouse model reproducing the pathophysiology of neonatal group B streptococcal infection
Source: Nat Commun. 2018 Aug 7;9:3138. doi: 10.1038/s41467-018-05492-y (PMC6081475; doi:10.1038/s41467-018-05492-y)
Supplement: Supplementary file 1 — Supplementary Information [file 41467_2018_5492_MOESM1_ESM.pdf]

a

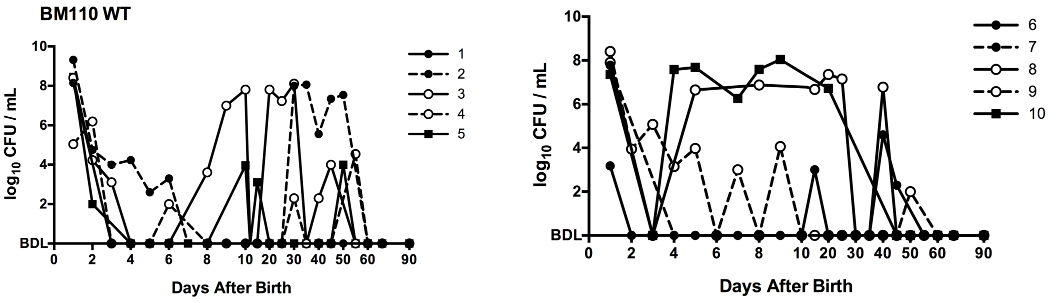

b

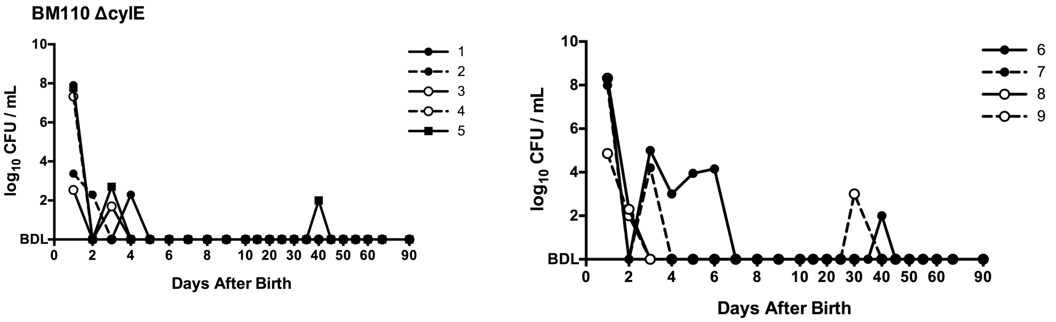

Supplementary Fig. 1 | Vaginal GBS colonisation of individual female mice. Pregnant BALB/c mice were intra-vaginally inoculated with $3x10^4$ CFU of GBS BM110 WT or BM110ΔcylE at gestational days 17 and 18. **a-b** Upon birth, the number of GBS colony-forming units (CFU) in vaginal tract was determined at indicated time-points by enumerating BM110 WT- (**a**) or BM110ΔcylE- (**b**) in vaginal washouts of colonised progenitors. Each curve represents data for an individual mouse [n=10 (BM110 WT) and n=9 (BM110ΔcylE)].

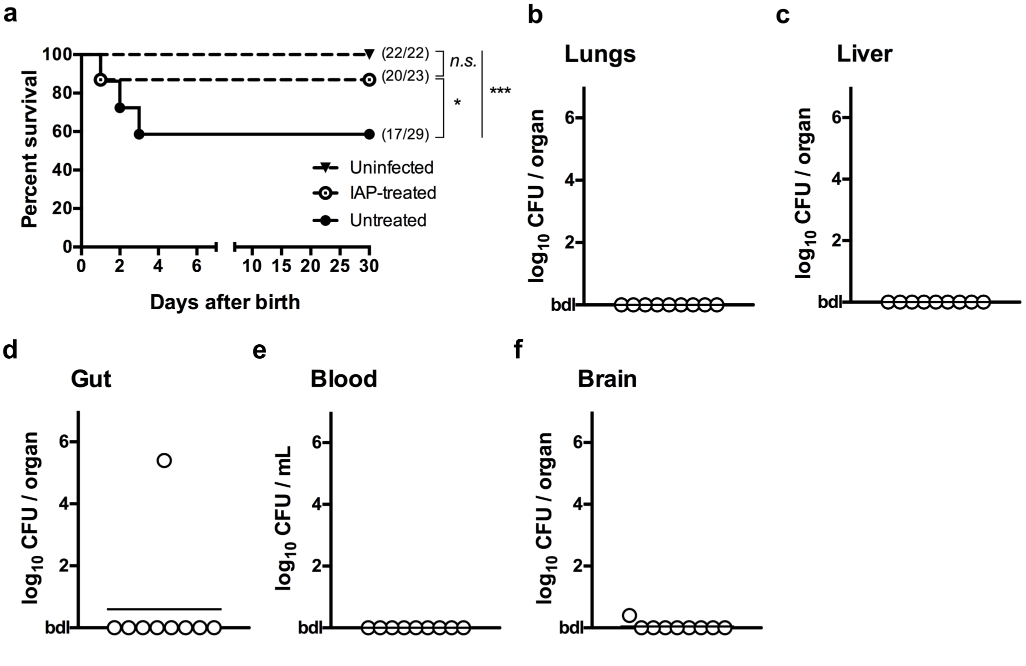

Supplementary Fig. 2 | IAP treatment leads to reduced vertical transmission of GBS. Pregnant BALB/c mice were intra-vaginally inoculated with $3x10^4$ CFU of BM110 WT or PBS (uninfected) at gestational days 17 and 18. At G20, infected females were given antibiotics in their drinking water (IAP-treated) or left untreated (Untreated). Uninfected dams were left untreated. **a** Kaplan-Meier survival curve of neonatal mice born from infected dams, IAP-treated or IAP-untreated, and uninfected dams monitored during a 30-days period. The numbers in parentheses represent the number of pups that survive versus the total number of pups born. Results represent data pooled from three (IAP-treated) or four (untreated) independent experiments [n=22 (uninfected), n=23 (IAP-treated) and n=29 (untreated)]. Comparisons with log-rank (Mantel-Cox) test. *$P < 0.05$, ***$P < 0.001$ and n.s., not significant. **b-f** GBS counts in the lungs (**b**), liver (**c**), gut (**d**), blood (**e**), and brain (**f**) of offsprings from IAP-treated progenitors at PND5. Each symbol indicates the data from a single pup (mean, n=9).

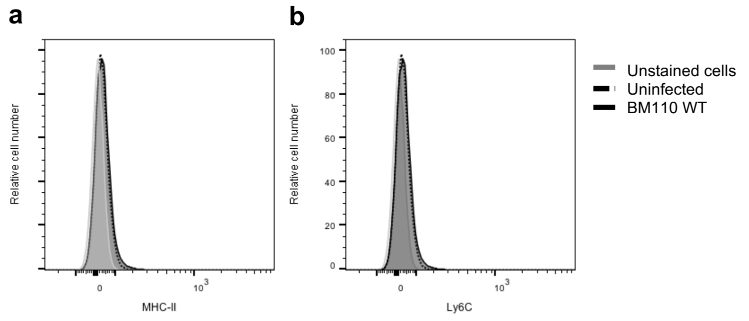

Supplementary Fig. 3 | Prenatal-GBS infection effect on microglia. Pregnant BALB/c mice were intra-vaginally inoculated with 3x10^4 CFU of GBS BM110 WT or PBS (uninfected) at gestational days 17 and 18. Flow cytometry of isolated leukocytes was performed at PND5. **a-b** Representative histograms showing surface expression of the markers MHC-II (**a**) and Ly6C (**b**) on microglia. Black line, pups infected with BM110 WT; dotted line, uninfected pups; grey line, unstained cells.

Supplementary Table 1 | Amino acid neurotransmitter concentrations in hippocampus, thalamus, cerebellum and prefrontal cortex in mice that survived to neonatal GBS infection, or uninfected controls, at PND90. Related to Fig. 5.

Neurotransmitter	*Group*	*Hippocampus*	*Thalamus*	*Cerebellum*	*Prefrontal cortex*
Glutamate	Uninfected	---*	---*	0.571 ± 0.0705	0.467 ± 0.0851
	BM110 WT-Survivors	---*	---*	0.437 ± 0.117	0.567 ± 0.0998
GABA	Uninfected	0.0574 ± 0.0199	0.0286 ± 0,00709	0.00853 ± 0.00168	0.0123 ± 0.00231
	BM110 WT-Survivors	0.0378 ± 0.0133	0.0252 ± 0,00525	0.0112 ± 0.00333	0.00723 ± 0.00280

*, Data represented in Figure 5.
Data represent amino acid levels in the different brain regions of the different experimental groups, and are expressed as mg per mg total protein, and represent mean ± SEM, from 6 mice per group.

Supplementary Table 2 | Monoamines neurotransmitter concentrations in hippocampus, striatum, prefrontal cortex and amygdala in mice that survived to neonatal GBS infection, or uninfected controls, at PND90. Related to Fig. 5.

Neurotransmitter	*Group*	*Hippocampus*	*Striatum*	*Prefrontal cortex*	*Amygdala*
NE	Uninfected	0.220 ± 0.0464	13.474 ± 2.724	0.733 ± 0.0575	20.170 ± 2.162
	BM110 WT-Survivors	0.125 ± 0.0136	15.775 ± 1.386	1.222 ± 0.220	15.203 ± 1.493
DA	Uninfected	---*	---*	4.973 ± 1.795	30.686 ± 6.241
	BM110 WT-Survivors	---*	---*	10.59 ± 4.312	27.581 ± 4.825
DOPAC	Uninfected	---*	---*	2.300 ± 0.785	8.300 ± 1.923
	BM110 WT-Survivors	---*	---*	3.070 ± 1.134	6.843 ± 1.841
HVA	Uninfected	BDL	---*	1.912 ± 0.454	7.059 ± 1.871
	BM110 WT-Survivors	BDL	---*	1.700 ± 0.365	11.842 ± 2.217

BDL, below detection limit
*, Data represented in Figure 5.
Data represent several monoamines neurotransmitters levels in the different brain regions of the different experimental groups, and are expressed as ng per mg total protein, and represent mean ± SEM, from 6 mice per group.
